# Molecular mechanisms of skeletal muscle fibrosis and potential targeted therapeutic strategies

**DOI:** 10.3389/fimmu.2026.1714238

**Published:** 2026-01-30

**Authors:** Jiahuan Gong, Jingxuan Xu, Jitai Zhang, Yuntian Shen, Hualin Sun, Bingqian Chen

**Affiliations:** 1 Jiangsu Key Laboratory of Tissue Engineering and Neuroregeneration, Co-innovation Center of Neuroregeneration, Nantong University, Nantong, Jiangsu, China; 2 Key Laboratory of Neuroregeneration of Ministry of Education, Co-innovation Center of Neuroregeneration, Nantong University, Nantong, Jiangsu, China; 3 Department of Orthopedics, Changshu Hospital Affiliated to Soochow University, First People’s Hospital of Changshu City, Changshu, Jiangsu, China

**Keywords:** anti-fibrotic therapy, FAPs, skeletal muscle fibrosis, TGF-β, YAP/TAZ

## Abstract

Skeletal muscle fibrosis is a pathological process characterized by excessive deposition of extracellular matrix (ECM). It commonly occurs in various diseases such as muscular dystrophy, aging, cancer cachexia, and muscle injury. This condition leads to destruction of muscle structure, loss of function, and impaired regeneration, significantly affecting patients’ quality of life. This review systematically summarizes the molecular mechanisms underlying skeletal muscle fibrosis. Key signaling pathways include transforming growth factor-beta (TGF-β)/Smad, yes-associated protein/transcriptional coactivator with PDZ-binding motif (YAP/TAZ), inflammation and immune regulation, oxidative stress, and microRNA-mediated regulation. The roles of fibro/adipogenic progenitors (FAPs), macrophages, and myofibroblasts in this process are also discussed. Among these, the TGF-β/Smad pathway acts as a central driver of fibrosis by promoting the differentiation of FAPs into myofibroblasts and stimulating ECM synthesis. YAP/TAZ integrates mechanical and biochemical signals, further amplifying the fibrotic response. Inflammation, oxidative stress, and epigenetic regulators such as miRNAs and lncRNAs also contribute through complex networks. Regarding therapeutic strategies, this article highlights various interventions including pharmacological inhibition (e.g., TGF-β inhibitors, angiotensin-converting enzyme inhibitors/angiotensin II receptor blockers (ACEIs/ARBs), antioxidants), gene- and RNA-targeting therapies (e.g., miRNA mimics or inhibitors), cell-based therapies (e.g., Mesenchymal Stem Cells (MSCs)), biological agents (e.g., anti-connective tissue growth factor (CTGF) antibodies), as well as physical and nutritional interventions (e.g., electroacupuncture, magnetic stimulation, natural compounds). These approaches demonstrate strong anti-fibrotic potential by modulating ECM metabolism, the immune microenvironment, and cellular behaviors. However, current research still faces challenges such as disease heterogeneity, optimal treatment timing, drug delivery issues, and long-term safety concerns. Therefore, future studies should focus on developing highly specific targeted therapies, integrating multi-omics technologies and imaging assessments, and advancing personalized combination strategies to ultimately achieve effective prevention and treatment of skeletal muscle fibrosis.

## 1 Introduction

Skeletal muscle fibrosis is characterized by excessive deposition of extracellular matrix, primarily driven by abnormal accumulation of collagen. This leads to the progressive replacement of normal muscle fibers with fibrotic scar tissue, thereby disrupting muscle architecture, diminishing elasticity, and causing severe functional impairment. This pathological condition is commonly observed in various muscle-related disorders. Examples include hereditary muscular dystrophies such as Duchenne muscular dystrophy (DMD), age-related sarcopenia, cancer cachexia, abnormal repair after injury, and chronic metabolic diseases like diabetic myopathy (1–3). As the disease progresses, fibrosis not only reduces muscle strength, limits joint mobility, and impairs motor function, but also hampers muscle regeneration. It significantly affects patients’ quality of life and may increase the risk of reinjury and complications (4). Therefore, understanding the underlying mechanisms and developing effective intervention strategies are of great clinical importance.

From a pathophysiological perspective, skeletal muscle fibrosis is not a standalone disease but rather a common terminal pathway resulting from various etiologies. Its development involves alterations in multiple cellular behaviors and dysregulation of molecular pathways. These include abnormal activation and differentiation of fibroadipogenic progenitors (FAPs), infiltration and polarized imbalance of inflammatory cells, elevated oxidative stress, and aberrant activation of intracellular signaling cascades such as TGF-β/Smad and YAP/TAZ. These processes interact intricately, creating a pro-fibrotic microenvironment that further accelerates ECM deposition and tissue architectural disruption (5, 6). Elucidating the interactions among these mechanisms is crucial for identifying key therapeutic targets.

Critically, the immune system acts as a primary orchestrator of skeletal muscle fibrosis, bridging initial tissue damage to the sustained activation of fibrogenic pathways. Dysregulated immune responses—characterized by persistent inflammation, failed resolution, and maladaptive communication with muscle-resident stromal cells—establish a self-perpetuating profibrotic microenvironment. This immune-centric perspective positions immune cells not as passive contributors but as dynamic regulators that dictate the balance between successful regeneration and pathological scarring (7, 8) Therefore, targeting these immune-driven circuits and restoring physiological immune-stromal crosstalk emerges as a promising therapeutic strategy to halt fibrosis and promote genuine muscle repair.

Recent advances in research techniques, particularly the widespread application of single-cell transcriptomic sequencing, spatial multi-omics technologies, and bioinformatic analytical methods, have deepened our understanding of cellular heterogeneity, molecular regulatory networks, and microenvironmental crosstalk in skeletal muscle fibrosis. These approaches have uncovered previously unknown cell subpopulations, such as specific macrophage subtypes and activated FAPs, and have identified multiple potential therapeutic targets. These findings provide a theoretical foundation for developing precise intervention strategies (9, 10). Future research is expected to advance anti-fibrotic treatments toward individualized and multi-target combination therapies. Although various anti-fibrotic strategies—including pharmacological inhibition, genetic regulation, cell-based therapies, and physical interventions—have demonstrated efficacy in animal models, their translation into clinical practice remains challenging. Key obstacles include disease heterogeneity, narrow therapeutic time windows, low drug delivery efficiency, and long-term safety concerns (11, 12). Therefore, systematically elucidating the molecular mechanisms of skeletal muscle fibrosis and summarizing recent therapeutic advances are crucial for identifying translationally promising strategies.

This review aims to comprehensively summarize the core pathogenic mechanisms of skeletal muscle fibrosis, including key signaling pathways, cell types, and their interactions. It also discusses both existing and emerging therapeutic strategies. Finally, future research directions in this field are proposed to provide insights for related basic research and clinical treatment.

## 2 Molecular mechanisms of skeletal muscle fibrosis

### 2.1 Overview of pathophysiological sequence

**Figure 1:**
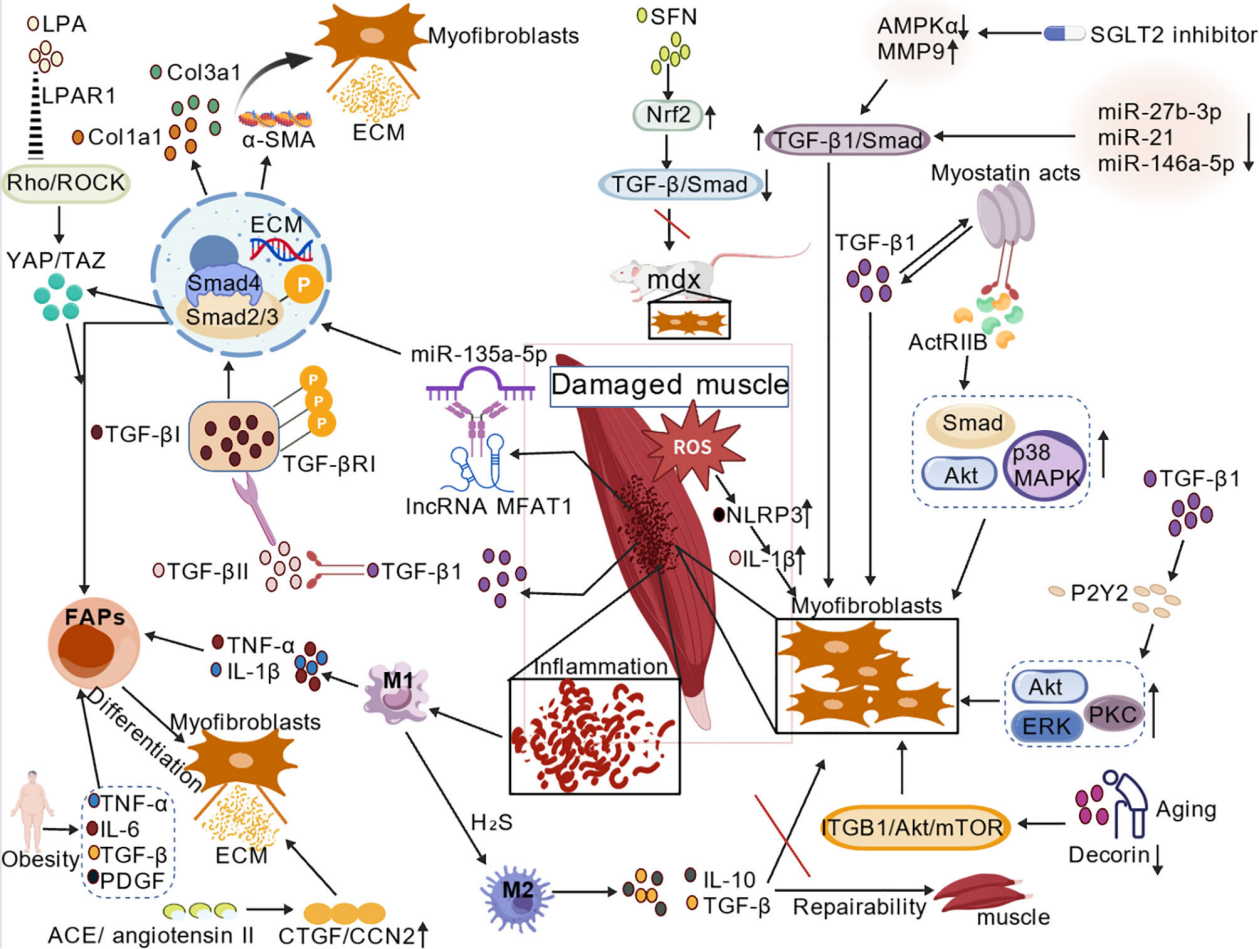
Overview of the molecular mechanisms of skeletal muscle fibrosis. This figure summarizes the key signaling pathways and molecular regulatory networks involved in skeletal muscle fibrogenesis. Major mechanisms include: activation of the canonical TGF-β/Smad pathway and its crosstalk with the YAP/TAZ mechanosignaling pathway; fibrosis promotion through inflammatory and immune responses, such as macrophage polarization and oxidative stress; epigenetic regulation of fibrotic gene expression by miRNAs and lncRNAs; as well as the involvement of other pathways including Myostatin. Together, these processes drive excessive ECM deposition and lead to loss of muscle function.

Skeletal muscle fibrosis is the pathological endpoint of failed regeneration, characterized by excessive and irreversible deposition of ECM, primarily collagens. This process initiates with muscle damage, which triggers an acute inflammatory response involving the infiltration of neutrophils and pro-inflammatory (M1) macrophages that clear debris. This phase is followed by a reparative stage dominated by anti-inflammatory (M2) macrophages and regulatory T cells (Tregs). A critical determinant of the outcome is the fate of resident mesenchymal stem cells known as FAPs. In successful regeneration, FAPs support myogenesis and are subsequently cleared. Under persistent inflammatory and profibrotic signaling, however, FAPs proliferate and differentiate into ECM-secreting myofibroblasts, the principal effector cells of fibrosis. Key soluble factors driving this pathological transition include TGF-β, Connective Tissue Growth Factor (CTGF/CCN2), Platelet-Derived Growth Factor (PDGF), and components of the Renin-Angiotensin System (RAS). The altered ECM itself, enriched in fibrinogen, fibronectin, and decorin, creates a stiffened microenvironment that further promotes myofibroblast activation via mechanosensitive pathways like YAP/TAZ. The balance between matrix synthesis, governed by TGF-β, and its degradation, controlled by Matrix Metalloproteinases (MMPs) and their inhibitors (TIMPs), is profoundly disrupted. This complex, multicellular network converges to replace functional contractile tissue with non-compliant fibrotic scar, leading to impaired muscle function and regeneration. The following sections detail the core signaling pathways that orchestrate this deleterious process (**Figure 1**).

### 2.2 TGF-β as the central driver of fibrosis

The canonical TGF-β/Smad pathway is the master regulator of fibrosis across tissues. Macrophage-derived TGF-β is a principal activator of the canonical Smad pathway in FAPs, directly linking early immune responses to fibrotic commitment (7, 13). In skeletal muscle, its activation is particularly consequential for the fate determination of FAPs. FAPs are the major cellular source of fibrotic ECM in muscle diseases. TGF-β, often released by infiltrating immune cells and damaged myofibers, acts as a potent instructor of FAP differentiation.

#### 2.2.1 Canonical activation of the TGF-β/Smad signaling pathway

During skeletal muscle injury or disease, TGF-β1 becomes highly activated and released. It binds to the TGF-β type II receptor (TGF-βRII) on the cell membrane, leading to phosphorylation of Smad2/3. Phosphorylated Smad2/3 complexes with Smad4 and translocates to the nucleus, where it drives transcription of pro−fibrotic genes such as collagen I, collagen III, α−SMA, and fibronectin. There, it acts as a transcription factor by directly binding to promoter regions of specific genes such as those related to collagen and extracellular matrix (14). This process significantly upregulates the expression of multiple pro-fibrotic genes including collagen I (Col1a1), collagen III (Col3a1), α-smooth muscle actin (α-SMA), and fibronectin. Consequently, it drives the synthesis and deposition of ECM. Thus, the TGF-β1/Smad signaling pathway plays a central regulatory role in skeletal muscle fibrosis.

#### 2.2.2 Regulation of FAP fate

FAPs are resident mesenchymal stromal cells in skeletal muscle that play a dual role in tissue homeostasis and pathology. Under physiological conditions, FAPs support muscle regeneration by creating a permissive microenvironment for satellite cells (15). Seminal work by Rossi and colleagues established FAPs as the primary cellular source of ECM-producing myofibroblasts in fibrotic muscle diseases (16, 17). Under pathological conditions with sustained TGF-β activation, FAPs are abnormally activated through Smad2/3 phosphorylation. This leads to their differentiation into myofibroblasts that secrete excessive ECM, making them a major cellular source of fibrosis (5, 18). This fundamental concept, established through foundational research on FAP biology, underscores their targeting as a central therapeutic strategy. Furthermore, TGF-β signaling in FAPs is modulated by crosstalk with other pathways; for instance, PDGFRα activation synergizes with TGF-β to drive fibrotic commitment (19). A 2025 study further revealed that TGF-β activates the mechanosensitive co-transcriptional regulators YAP/TAZ via the canonical Smad3 pathway. These factors form a synergistic mechanism that ultimately determines the differentiation fate of FAPs into myofibroblasts (5). Inhibition of FAP proliferation or promotion of their timely apoptosis is crucial for preventing excessive ECM accumulation, highlighting FAPs as a central therapeutic target in muscle fibrosis (20, 21). This mechanism provides a new theoretical basis and potential intervention strategies for targeting fibrotic diseases.

Furthermore, FAP activation and fate are profoundly regulated by the immune microenvironment. Macrophages are pivotal in this regulation. Pro-inflammatory (M1) macrophages secrete cytokines like TNFα and IL-1β that can prime FAPs, while anti-inflammatory (M2) macrophages, particularly through TGF-β1 secretion, directly drive their differentiation into ECM-producing myofibroblasts (7, 22). Recent single-cell studies have identified specific macrophage subpopulations (e.g., Spp1+ or Galectin-3+ macrophages) that are enriched in fibrotic niches and exhibit potent FAP-stimulatory activity (13, 23). Beyond macrophages, type 2 innate lymphoid cells (ILC2s) and eosinophils contribute to fibrotic signaling; ILC2-derived IL-5 and IL-13, along with IL-33 from stromal cells, can modulate FAP function and promote a profibrotic environment, as seen in muscular dystrophy (24). The complement system also engages FAPs; for instance, C3a derived from FAPs can recruit and modulate monocytes/macrophages, creating a feed-forward loop that exacerbates fibrosis (22). Thus, FAPs are not autonomous effectors but are critically instructed by a complex network of immune signals.

The advent of single-cell transcriptomics has revolutionized our understanding of FAP biology, revealing substantial heterogeneity within this population. Distinct FAP subsets exhibit differential responses to fibrotic cues. For instance, a specific FAP subpopulation characterized by high expression of Pdgfra and Ccn2 is highly responsive to TGF-β and LPA signaling, and is markedly expanded in fibrotic muscle from dystrophic mice (4, 25). This subset shows upregulated expression of ECM genes and pathways related to collagen biosynthesis and organization. Single-cell analyses have also mapped the cellular communication networks in fibrotic muscle, identifying FAPs as central hubs that receive signals from macrophages (e.g., via TGFB1-TGFBR, CCL2-CCR2) and, in turn, secrete factors like CCN2/CTGF that further amplify the fibrotic response (9, 25). These findings underscore the importance of targeting specific pathogenic FAP subsets for anti-fibrotic therapy.

### 2.3 Other key fibrogenic mediators and pathways

The core mechanism of skeletal muscle fibrosis involves excessive deposition of ECM, primarily mediated by the activation of myofibroblasts. This process is regulated by multiple molecules through complex networks. Myostatin acts as a key pro-fibrotic factor. It binds to activin receptor IIB (ActRIIB) and activates Smad, p38 MAPK, and Akt signaling pathways. This directly induces the proliferation of muscle fibroblasts and the synthesis of ECM proteins (26). Moreover, Myostatin forms a positive feedback loop with TGF-β1, mutually promoting each other’s expression and collectively exacerbating fibrosis (27–29). On the other hand, the purinergic receptor P2Y2 also plays an important role in fibrosis. Under TGF-β1 stimulation, P2Y2 activates Akt, ERK, and PKC signaling pathways. This promotes fibroblast proliferation, migration, and differentiation into myofibroblasts, while enhancing the production of ECM components such as collagen (30). These findings highlight its critical role in the fibrotic signaling network. Decorin, a small leucine-rich proteoglycan component of the ECM, exerts a sarcoprotective role. Its deficiency, as seen in aging, exacerbates skeletal muscle fibrosis by dysregulating the ITGB1/Akt/mTOR signaling pathway, highlighting the importance of ECM composition in modulating fibrotic responses (31). Meanwhile, the RAS is also deeply involved in fibrosis regulation. Key components such as angiotensin-converting enzyme (ACE) and angiotensin II significantly promote ECM accumulation by upregulating CTGF/CCN2 expression. ACE inhibitors like enalapril effectively alleviate fibrosis in models of muscular dystrophy and diabetes. This effect is closely linked to the specific inhibition of CTGF rather than TGF-β1 (32, 33), suggesting that targeting the RAS/CTGF axis holds clear therapeutic potential. In summary, interventions targeting Myostatin, P2Y2, and the ACE/CTGF signaling pathways provide important theoretical and experimental support for multi-target treatment strategies against skeletal muscle fibrosis.

### 2.4 Convergent intracellular signaling pathways

The TGF-β/Smad signaling pathway does not function in isolation. It interacts with multiple other signaling pathways to form a complex network that collectively amplifies fibrotic responses (34, 35). This crosstalk further underscores the central role of this pathway in fibrosis. The AMPK/MMP9 axis represents another regulatory mechanism. Studies show that the SGLT2 inhibitor empagliflozin exerts anti-fibrotic effects by activating AMPKα and inhibiting MMP9, thereby negatively regulating the TGF-β1/Smad pathway (36). This mechanism provides a new theoretical basis for metabolic interventions in fibrosis. Multiple miRNAs serve as critical negative feedback regulators of the TGF-β/Smad pathway. For instance, miR-27b-3p directly targets TGF-βR1 mRNA and suppresses its expression, thereby inhibiting Smad2/3 phosphorylation and alleviating fibrosis (18). Similarly, miR-21 enhances pro-fibrotic signaling by targeting inhibitory Smad7 and relieving its suppression on Smad2/3 (37). Meanwhile, miR-146a-5p inhibits the entire pathway by directly targeting Smad4 (38). These findings highlight the therapeutic potential of miRNAs in fibrosis regulation. Oxidative stress and chronic inflammatory conditions enhance TGF-β/Smad pathway activity. For example, sulforaphane (SFN) activates the Nrf2-mediated antioxidant response and inhibits TGF-β/Smad signaling, thereby reducing muscle fibrosis in the mdx mouse model of muscular dystrophy (39). This suggests that anti-inflammatory and antioxidant strategies may represent important approaches to mitigate fibrosis. Notably, TGF-β not only acts directly on fibroblasts but also promotes fibrosis indirectly by regulating other factors such as CTGF/CCN2 (32, 40). Importantly, work by Rebolledo DL has elucidated the critical TGF-β/CTGF axis in fibrosis, demonstrating that CTGF can drive fibrosis independently of TGF-β in certain contexts, such as denervation (41). Furthermore, TGF-β activates non-Smad pathways including p38 MAPK and Akt, which collaboratively drive fibrotic progression (42). Therefore, targeting multiple pathways simultaneously may be a key strategy for future therapies.

### 2.5 YAP/TAZ mechanical signaling pathway

YAP and TAZ are key transcriptional co-activators that integrate biochemical and mechanical signals in the regulation of muscle fibrosis. The crosstalk between TGF-β and YAP/TAZ is bidirectional and synergistic. TGF-β can activate YAP/TAZ via its canonical Smad3 pathway independently of the Hippo kinase cascade (5). Conversely, nuclear YAP/TAZ can complex with Smad2/3 to enhance the transcription of TGF-β target genes such as CTGF and plasminogen activator inhibitor-1 (PAI-1), establishing a potent pro-fibrotic feedback loop (5, 41). This interaction positions YAP/TAZ not merely as a parallel pathway but as an integral co−transcriptional amplifier of TGF−β−driven fibrogenesis in FAPs, forming a core mechano−chemical signaling circuit in fibrosis.

Mechanical signaling actively contributes to YAP/TAZ activation. Increased muscle stiffness or upregulation of the Rho/ROCK pathway promotes nuclear translocation and transcriptional activity of YAP/TAZ. Conversely, inhibiting mechanical signaling—using the Rho inhibitor C3 or culturing cells on soft substrates—or administering the YAP/TAZ inhibitor verteporfin significantly suppresses TGF−β1−mediated fibrogenic differentiation of FAPs (5). Beyond mechanical cues, biochemical signals such as lysophosphatidic acid (LPA) also potently activate YAP/TAZ. LPA signals through LPAR1, activating the downstream Rho/ROCK pathway, which is particularly significant in FAPs. Recent single−cell RNA sequencing studies reveal that LPA signaling acts as a key driver shifting specific FAP subpopulations toward a proliferative and pro−fibrotic transcriptional program (20, 43), promoting expression of pro−fibrotic factors like CCN2/CTGF and enhancing FAP migratory capacity (44, 45). Notably, age−related stiffening of the muscle matrix can also trigger fibrosis through YAP/TAZ activation (46). Collectively, these findings underscore the pivotal role of YAP/TAZ as signaling hubs that converge biochemical and mechanical inputs to drive fibrosis. Interventions targeting YAP/TAZ or associated mechanotransduction pathways may therefore offer promising therapeutic strategies for muscle fibrotic diseases.

Beyond matrix stiffness, immune cells actively modulate YAP/TAZ activity in fibrotic muscle. Pro-inflammatory M1 macrophages secrete cytokines such as TNF-α and IL-1β, which can induce cytoskeletal remodeling and Rho/ROCK activation in neighboring FAPs and fibroblasts, thereby promoting YAP/TAZ nuclear translocation (9, 10, 47). Conversely, TGF-β released from M2 macrophages not only activates Smad signaling but also synergizes with mechanical cues to potentiate YAP/TAZ-mediated transcription of pro-fibrotic genes like CTGF and PAI-1 (5, 41). Furthermore, macrophage-derived lysophosphatidic acid (LPA) signals through LPAR1 on FAPs, activating Rho/ROCK and YAP/TAZ to drive fibrogenic differentiation and migration (44, 45). Thus, YAP/TAZ serves as a signaling hub that integrates both immune cytokine cues and biomechanical inputs to coordinate fibrotic responses.

### 2.6 Inflammation, immune dysregulation, and fibrosis

**Figure 2:**
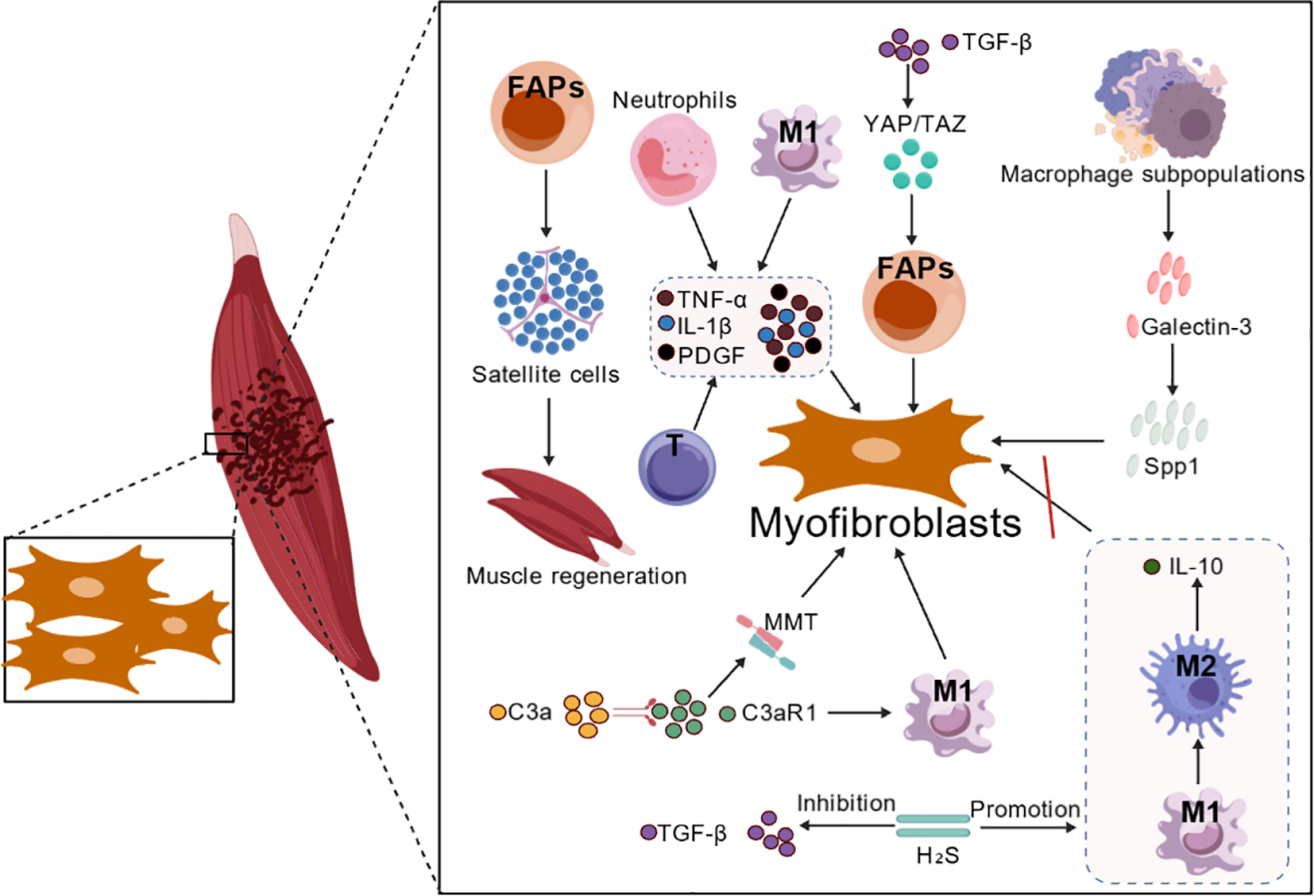
Cellular interactions in the fibrotic niche. This figure depicts the major cell populations contributing to skeletal muscle fibrosis. Key players include fibro-adipogenic progenitors (FAPs), which differentiate into myofibroblasts driven by TGF-β and YAP signaling; macrophages (M1/M2) that modulate inflammation and fibrosis via cytokine release; other immune cells (such as T cells and neutrophils) along with the complement system, which participate in microenvironment remodeling; as well as endothelial cells and satellite cells, both indirectly influencing the fibrotic process through angiogenesis and regenerative functions.

The immune system is a central conductor of skeletal muscle fibrosis, coordinating the transition from acute injury to chronic fibrotic pathology. This process involves a tightly regulated but often dysregulated crosstalk between infiltrating immune cells (e.g., macrophages, T cells, neutrophils) and tissue-resident cells (e.g., FAPs, satellite cells). A failure in immune resolution—marked by a prolonged pro-inflammatory phase and impaired transition to a pro-regenerative state—is a hallmark of fibrotic progression (48, 49). The following sections detail how specific immune cells and signaling modules drive this deleterious outcome (**Figure 2**).

Chronic inflammation initiates the fibrotic process. It is one of the primary triggers of skeletal muscle fibrosis. In obesity, pro-inflammatory cytokines such as TNF-α and IL-6, as well as lipotoxic substances secreted by adipose tissue, can induce systemic low-grade inflammation and promote immune cell infiltration into skeletal muscle, including macrophages and T cells (50, 51). These immune cells further activate FAPs by releasing factors such as TGF-β and PDGF, thereby promoting collagen synthesis and abnormal deposition. This highlights the central role of inflammation in initiating fibrosis. Moreover, oxidative stress, for example via NOX2/4-mediated ROS generation, forms a positive feedback loop with chronic inflammation, mutually exacerbating both processes and further driving fibrosis progression (52, 53). This mechanism not only amplifies initial inflammatory signals but also results in excessive accumulation of ECM components. Therefore, targeting the crosstalk between inflammation and oxidative stress may offer new strategies for anti-fibrotic therapy.

Immune cell polarization regulates fibrosis. Macrophages are key immune cells controlling fibrosis. The temporal switch from M1 to M2 polarization is crucial for directing FAP function. While timely M2 activity supports regeneration, sustained or dysregulated M2 signaling, often via TGF-β1, directly instructs FAPs to adopt a profibrotic myofibroblast fate (7, 13). Conversely, immune-mediated clearance of FAPs via apoptosis is essential for resolving their transient supportive role, and defects in this clearance mechanism contribute to FAP accumulation and fibrosis (49). M1 macrophages (CD68^+^) dominate the early inflammatory phase and secrete pro-inflammatory factors such as TNF-α and IL-1β, which directly damage muscle fibers and activate FAPs. In contrast, M2 macrophages (CD206^+^) promote tissue repair and fibrosis through the secretion of IL-10 and TGF-β (47). The balance between these two subsets critically influences the fibrotic process. In injury models, exogenous H_2_S can promote the transition from M1 to M2 phenotype and suppress the TGF-β signaling pathway, thereby significantly reducing collagen deposition (52). This supports targeting macrophage polarization as a potential therapeutic strategy to alleviate fibrosis. Additionally, Tregs may indirectly modulate fibrosis by suppressing excessive immune responses. Further investigation into the interaction between Tregs and macrophages may provide new directions for immune-based regulation of fibrotic diseases.

Crosstalk among key signaling pathways drives fibrosis. The TGF-β/Smad pathway is a core driver of fibrosis. TGF-β1, released by immune cells and damaged muscle fibers, promotes collagen gene expression such as COL1A1 through Smad2/3 phosphorylation and inhibits MMP activity, leading to ECM metabolic imbalance (47). Immune-mediated tissue stiffening—through deposition of provisional matrices rich in fibrinogen and fibronectin—creates a mechanically altered microenvironment that further activates YAP/TAZ in resident mesenchymal cells, establishing a feed-forward loop between inflammation and mechanotransduction (5, 46). Thus, this pathway is considered a key signaling axis regulating fibrosis. The TWEAK/NF-κB pathway is highly activated in chronic spinal cord injury and promotes fibrosis by enhancing inflammatory cytokine expression and fibroblast proliferation (54). This mechanism, together with the TGF-β/Smad pathway, forms an important molecular basis for fibrosis. The RAS also participates in fibrosis regulation. Ang-II promotes fibrosis via the AT1 receptor, while Ang-(1-7) counteracts this effect through the Mas receptor and enhances muscle regeneration (55). This indicates that different components of the RAS system exert opposing effects in fibrosis, offering potential therapeutic targets.

Oxidative stress and inflammation synergistically promote fibrosis. These processes are closely linked and collectively drive fibrotic progression. In aging or metabolic diseases, ROS activate the NOD-like receptor protein 3 (NLRP3) inflammasome to promote IL-1β secretion and enhance TGF-β signaling (51). For example, highland barley tea polyphenols (HBP) can reduce collagen deposition by upregulating SIRT3 expression and suppressing oxidative stress and inflammatory responses (56). Similarly, hydrogen sulfide (H_2_S) treatment alleviates oxidative damage and fibrosis by reducing the expression of gp91phox, a NOX2 subunit (52). These findings highlight the potential value of targeting oxidative stress and inflammatory pathways in fibrosis treatment.

In summary, inflammatory and immune signaling pathways drive skeletal muscle fibrosis through multi-level mechanisms including cell infiltration, cytokine secretion, and oxidative stress. Targeting these pathways, such as TGF-β, macrophage polarization, and ROS, may provide new therapeutic strategies. Future studies should further explore the interactions between immune cells and FAPs, as well as their clinical translational potential.

### 2.7 Adaptive immunity and immune memory in fibrosis

The immune system sustains persistent inflammation and fibrosis in muscle disorders through multiple interconnected pathways. Although regulatory T cells (Tregs) are important, other T cell subsets contribute significantly: pro-inflammatory Th1 and Th17 cells exacerbate chronic inflammation and fibrosis in myositis and muscular dystrophy, while cytotoxic CD8^+^ T cells directly injure muscle fibers (57). B cells and autoantibodies further play a key role, especially in autoimmune myopathies, where they target muscle or vasculature, activate complement, and perpetuate immune-driven fibrosis. Even in non-autoimmune dystrophies such as DMD, B cell infiltration appears to help sustain local inflammation. Crucially, the persistence of damage stems from a failure to resolve inflammation. Unlike a self-limiting, pro-resolving program—which involves mediators like resolvins and a shift from M1 to M2 macrophages—chronic inflammation is maintained by ongoing DAMPs/PAMPs, impaired efferocytosis, and a dysregulated cytokine environment. This failed resolution in turn activates fibrogenic cells. Emerging evidence points to “trained immunity”—a long-term functional reprogramming of innate immune cells such as macrophages after an initial stimulus—as a potential underpinning of this heightened inflammatory state, similar to observations in obesity or aging. This reprogramming involves metabolic changes, including increased glycolysis, which promotes a pro-fibrotic macrophage phenotype (23, 58). Together, these mechanisms highlight the immune system’s central role in driving fibrosis and suggest that targeting immune dysregulation may offer promising therapeutic strategies for muscle disorders.

### 2.8 Oxidative stress and aging-related pathways

Oxidative stress and aging are central drivers of skeletal muscle fibrosis, a condition marked by excessive ECM deposition—particularly collagen accumulation—that impairs muscle function and regeneration. The sustained oxidative stress associated with aging results from both increased ROS production and diminished antioxidant defenses. This not only directly damages cellular components but also activates a network of interconnected pro−fibrotic signaling pathways. Key pathways among these are the TGF−β/Smad, MAPK (p38/JNK), PI3K/Akt, and AMPK/mTOR cascades, which collectively amplify fibrotic responses. For instance, ROS upregulate TGF−β1 expression and enhance Smad2/3 phosphorylation, promoting myofibroblast differentiation and ECM synthesis (1, 59). Notably, immune cells are major sources of ROS in fibrotic niches. Macrophages, particularly pro-inflammatory M1 subsets, exhibit enhanced glycolytic metabolism and mitochondrial ROS production, which further amplifies TGF-β1 expression and activation in a paracrine manner, thereby linking immunometabolism to fibrogenic signaling (52, 60). Similarly, ROS directly activate p38 and JNK, increasing collagen gene expression (e.g., COL1A1, COL3A1) and synergizing with TGF−β signaling (59, 61). Meanwhile, sustained Akt activation under oxidative stress accelerates collagen deposition, while exercise−induced myokines such as irisin can modulate PI3K/Akt signaling to reduce fibrosis (62, 63). In contrast, AMPK acts as an energy sensor that inhibits mTOR, suppresses ECM synthesis, and promotes autophagy, thereby counteracting fibrosis (36, 64–66). Notably, these pathways do not operate in isolation; they form a coordinated network that perpetuates fibrotic degeneration, as evidenced in aging models where elevated ROS levels coincide with marked collagen deposition and mitochondrial dysfunction (59, 61). Targeting key molecules within this interconnected signaling web therefore holds significant therapeutic potential for mitigating muscle fibrosis.

### 2.9 Epigenetic and post-transcriptional regulation

Skeletal muscle fibrosis is a common pathological alteration following various muscle injuries and diseases, such as Duchenne muscular dystrophy, critical limb ischemia, acute contusion, and diabetic myopathy. It is characterized by excessive deposition of ECM, which disrupts normal muscle structure and function and ultimately leads to loss of muscle performance. Recent research has increasingly highlighted the central role of microRNAs (miRNAs) and epigenetic regulation in this process, offering new perspectives for understanding its molecular mechanisms and developing targeted therapeutic strategies.

MiRNAs are a class of endogenous short non-coding RNAs that finely regulate gene expression at the post-transcriptional level by binding to the 3′-untranslated region (UTR) of target mRNAs, leading to mRNA degradation or translational inhibition. Several miRNAs have been identified as key regulators in skeletal muscle fibrosis (67, 68). On one hand, certain miRNAs exert protective effects by inhibiting pro-fibrotic signaling. For instance, the miR-29 family is widely recognized as a key negative regulator of fibrosis. It directly targets multiple collagen-coding genes such as Col1a1 and Col3a1. Its downregulation in a chronic critical limb ischemia (CLTI) model promotes abnormal ECM deposition (10). Similarly, in TGF-β1-induced fibrosis, suppressed expression of miR-24-3p and miR-122-5p enhances targeting inhibition toward Smad2 and Tgfbr2, thereby amplifying the TGF-β/Smad signaling pathway and forming a positive feedback loop (69). In contrast, miR-146a-5p negatively regulates this pathway by directly targeting Smad4, and its overexpression significantly alleviates fibrosis (38). On the other hand, some miRNAs directly promote fibrotic progression. For example, in DMD, exosome-delivered miR-199a-5p induces fibroblast activation into myofibroblasts by suppressing caveolin-1, thereby exacerbating collagen secretion (70). In diabetic myopathy, upregulated miR-139-5p targets nicotinamide phosphoribosyltransferase (NAMPT), leading to mitochondrial dysfunction and triggering fibrosis (71). Intervention studies also show that miRNAs targeting AT1a can downregulate TGF-β1/Smad3 signaling and reduce muscle fibrosis in a hypertension model (11). Together, these studies demonstrate that miRNAs play complex and critical roles in skeletal muscle fibrosis and hold significant potential as therapeutic targets.

In addition to miRNA-mediated regulation, immune cells undergo lasting epigenetic modifications that perpetuate fibrotic responses. Macrophages exposed to fibrotic microenvironmental cues (e.g., TGF-β, ECM fragments) can develop epigenetic memory via histone modifications (H3K4me3, H3K27ac) and DNA methylation changes, leading to sustained pro-fibrotic gene expression upon re-stimulation—a phenomenon akin to trained immunity (9). These adaptations enhance secretion of fibrogenic cytokines (IL-1β, TGF-β) and promote M2-like polarization, thereby fueling chronic fibrosis.

Epigenetic regulation, particularly the ceRNA mechanism mediated by long non-coding RNAs, acts as a conductor in controlling miRNA function. These lncRNAs sponge miRNAs and prevent them from binding to target mRNAs, thereby indirectly upregulating gene expression. In a model of acute skeletal muscle contusion, lncRNA MFAT1 expression was significantly increased. It sponges miR-135a-5p and relieves inhibition on TGFBR2 and Smad4, activating the TGF-β/Smad pathway and promoting fibrosis (72). Similarly, lncRNA H19 is highly expressed in fibrotic muscle. It binds to miR-20a-5p and removes suppression on TGFBR2, enhancing TGF-β signaling and driving myofibroblast fibrosis (73). These ceRNA networks explain how pro-fibrotic signals are persistently amplified under pathological conditions through epigenetic mechanisms. In addition, classical epigenetic modifications such as DNA methylation may also influence expression by regulating the promoter activity of miRNAs or their target genes. This represents an important direction for future research. Overall, microRNAs and epigenetic mechanisms together form a sophisticated regulatory network. Some miRNAs, including miR-29b, miR-1/133a, and miR-24/122, act as inhibitors, while others such as miR-199a-5p promote fibrosis (74, 75). LncRNAs like MFAT1 and H19 amplify pro-fibrotic signals via the ceRNA mechanism. These findings have significant translational value (72, 76). Restoring inhibitory miRNAs, for example using miR-29b mimics, or blocking promotive miRNAs such as with miR-199a-5p inhibitors, are potential therapeutic strategies. Targeting ceRNA networks, like silencing lnc-MFAT1 or H19, may also interrupt pro-fibrotic signaling. Future studies should further validate these mechanisms in humans and develop efficient and specific *in vivo* delivery systems. This will enable targeted therapies against miRNAs and epigenetic regulators to become effective clinical tools for combating skeletal muscle fibrosis.

## 3 Therapeutic strategies for skeletal muscle fibrosis

**Figure 3:**
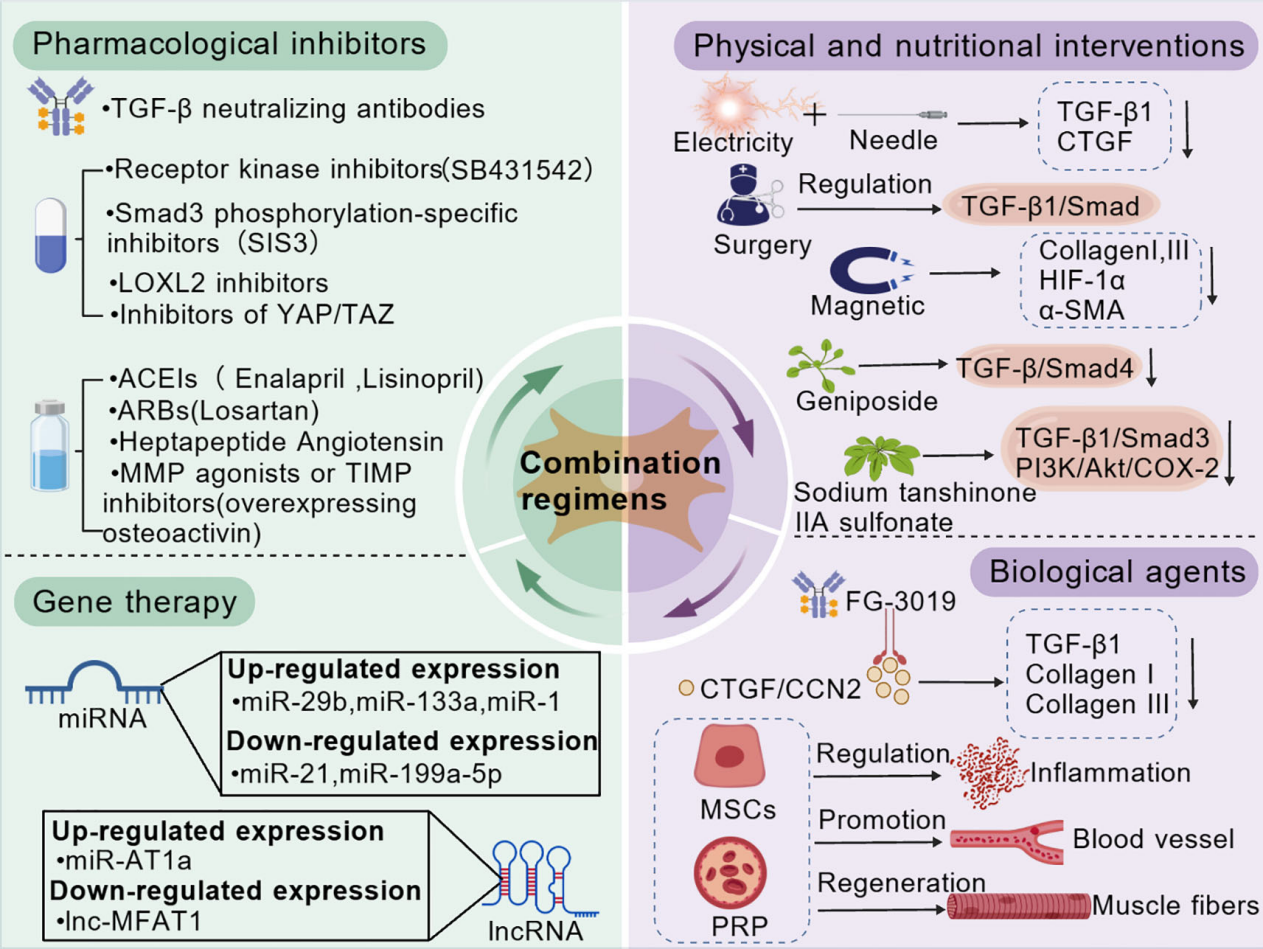
Landscape of therapeutic strategies for skeletal muscle fibrosis. This figure outlines current approaches to treat skeletal muscle fibrosis. Key strategies encompass: pharmacological inhibitors targeting pivotal molecules such as TGF-β, YAP/TAZ, and LOXL2; gene-based therapies employing miRNAs or lncRNAs; biological agents (e.g., anti-CTGF antibodies, MSCs, PRP); physical and nutritional interventions (including electroacupuncture, magnetic stimulation, and natural compounds); as well as combination regimens (for instance, TGF-β inhibitors combined with antioxidants). These interventions collectively aim to restore ECM metabolic balance, inhibit the activation of profibrotic cells, and enhance muscle regeneration.

Skeletal muscle fibrosis arises from a complex interplay of dysregulated signaling, aberrant cell activity, and microenvironmental dysfunction. Effective intervention requires a multi-pronged approach. This section reorganizes therapeutic strategies into a conceptual and translational framework, distinguishing between immunomodulatory approaches, muscle-intrinsic and metabolic interventions, and multimodal/combination strategies (**Figure 3**). We evaluate each based on mechanistic rationale, current evidence level, and translational considerations.

### 3.1 Targeting core fibrogenic signaling pathways

TGF-βserves as a key regulator in the fibrotic process. It promotes myofibroblast differentiation and excessive ECM synthesis through both Smad-dependent and Smad-independent signaling pathways. This ultimately leads to impairment of tissue structure and function. Thus, targeting the TGF-β signaling pathway has become a major research focus for anti-fibrotic therapy.

#### 3.1.1 Direct inhibition of the TGF-β signaling pathway

The TGF-β signaling pathway plays a critical role in skeletal muscle fibrosis. Its aberrant activation significantly promotes ECM deposition and impairs muscle contraction and regeneration (77). Studies indicate that directly inhibiting this pathway effectively mitigates fibrotic progression. Current strategies mainly include three types such as TGF-β neutralizing antibodies, receptor kinase inhibitors like SB431542, and Smad3 phosphorylation-specific inhibitors such as SIS3. These interventions potently block TGF-β/Smad signal transduction, reduce collagen synthesis and deposition, and suppress fibroblast activation (78, 79). Furthermore, in various animal models including ischemia-reperfusion injury and cancer cachexia, blocking TGF-β1 or its receptor not only markedly attenuates fibrosis but also promotes muscle regeneration and functional recovery (62, 80). These preclinical findings consistently demonstrate that directly targeting the TGF-β pathway represents a promising therapeutic strategy for skeletal muscle fibrosis with considerable translational potential.

#### 3.1.2 Inhibition of the RAS system

Overactivation of the RAS is closely linked to the development of fibrosis. By using angiotensin-converting enzyme inhibitors (ACEIs, such as Enalapril and Lisinopril) or angiotensin II receptor blockers (ARBs, such as Losartan), the binding of Ang II to its receptors is blocked, thereby indirectly suppressing downstream TGF-β/Smad signaling (32). This inhibition further reduces Smad2/3 phosphorylation levels and decreases fibroblast activation as well as excessive ECM accumulation (81). Notably, numerous animal studies and clinical investigations have confirmed that ACEIs and ARBs not only significantly reduce collagen content in muscle tissue but also improve muscle mechanical properties—such as grip strength and contractility—along with motor function (33). Thus, these inhibitors show promising therapeutic potential for treating fibrotic muscle disorders, including muscular dystrophy and chronic kidney disease-associated myopathy. Furthermore, the heptapeptide Angiotensin-(1-7), which acts via the Mas receptor to counterbalance Ang II effects, has shown promise in reducing fibrosis and improving muscle regeneration in dystrophic and radiation injury models (82, 83). Together, these findings highlight the therapeutic value of modulating the RAS pathway in mitigating fibrosis and enhancing muscle repair.

#### 3.1.3 Key regulatory mechanisms of the smad signaling pathway

The Smad signaling pathway plays a central role in TGF-β-mediated muscle fibrosis. Recent studies have revealed that microRNAs such as miR-24-3p and miR-122-5p can directly target Smad2 and TGF-βRII respectively. This action inhibits Smad2/3 phosphorylation and downstream pro-fibrotic gene expression, thereby exerting anti-fibrotic effects (69). On the other hand, tissue-nonspecific alkaline phosphatase (TNAP) is upregulated in pathologically hypertrophied hearts and dystrophic skeletal muscle. TNAP interacts with Smad2 protein and induces its dephosphorylation, thus negatively regulating the TGF-β signaling pathway and significantly mitigating fibrosis in both cardiac and skeletal muscle (84). Both mechanisms effectively suppress the fibrotic process by modulating the activity of the Smad2/3/4 complex. These findings offer molecular targets for developing new therapeutic strategies.

### 3.2 Modulation of immune and inflammatory responses

Immune and inflammatory responses play a central role in the development and progression of skeletal muscle fibrosis (6). This is particularly evident in macrophage polarization, complement system activation, and the cross-talk between oxidative stress and inflammatory factors. Together, these processes create a pro-fibrotic microenvironment. Targeting key steps in these pathways has become an important strategy for anti-fibrotic therapy. Macrophages play dual roles depending on their polarization states. Studies show that exogenous H_2_S promotes macrophage polarization toward the M2 anti-inflammatory phenotype. This alleviates local inflammation and suppresses collagen deposition, ultimately improving fibrosis after injury (52). Moreover, recent single-cell transcriptomic studies have identified two macrophage subpopulations closely associated with fibrosis. These subpopulations highly express Galectin-3 and secrete osteopontin (Spp1). They are enriched in fibrotic muscle areas and promote fibrosis by regulating the differentiation of matrix-producing precursor cells. Targeting these subsets can significantly delay fibrotic progression (9). Aberrant activation of the complement system also contributes to the fibrotic microenvironment. Especially after acute injury, the complement component C3a promotes macrophage-to-myofibroblast transition (MMT) via signaling through its receptor C3aR1. MMT is a key cellular source of fibrosis. Blocking the C3a receptor inhibits MMT and reduces muscle fibrosis (85). Several pharmacologic and nutritional interventions with anti-inflammatory and antioxidant properties show anti-fibrotic potential. Irisin activates the PI3K/Akt signaling pathway, reducing cellular senescence and oxidative stress while downregulating fibrosis-related proteins (62). The potent antioxidant astaxanthin alleviates immobilization-induced fibrosis by suppressing the TGF-β1 pathway and oxidative stress (86). Polyphenols extracted from highland barley tea (HBP) activate the deacetylase SIRT3, markedly improving age-related oxidative stress, inflammation, and fibrosis (56). Furthermore, natural phytochemicals such as quercetin and sulforaphane exhibit anti-fibrotic effects in multiple animal models. These effects involve inhibiting NOX2/4 activity, reducing ECM deposition, and modulating inflammatory cytokine expression. These findings suggest their potential as nutritional interventions (87). In summary, modulating immune and inflammatory responses is crucial for mitigating skeletal muscle fibrosis. Targeting macrophage polarization, the complement system, and the oxidative stress-inflammation network—particularly using natural compounds with anti-inflammatory and antioxidant activities—may offer novel strategies for the clinical prevention and treatment of fibrosis.

### 3.3 Regulation of ECM metabolism and remodeling

Restoring the balance between synthesis and degradation of the ECM is a key therapeutic strategy for skeletal muscle fibrosis. Excessive ECM deposition and increased collagen cross-linking are hallmarks of fibrosis, making interventions targeting ECM metabolism highly promising therapeutically. One approach involves modulating the balance between MMPs and TIMPs to promote collagen degradation and ECM remodeling. Studies show that using MMP agonists or TIMP inhibitors, such as overexpressing osteoactivin, significantly upregulates MMP-3 and MMP-9 expression, enhances collagen degradation, and reduces fibrosis (88). Similarly, electroacupuncture combined with massage therapy can regulate the MMP-1/TIMP-1 ratio, suppress abnormal ECM accumulation, and improve muscle structure and function (89). The TGF-β1/CTGF signaling pathway plays a central regulatory role in this process, and its inhibition can further normalize ECM metabolism (77). Another important antifibrotic strategy is to inhibit collagen cross-linking. Lysyl oxidase-like 2 (LOXL2) promotes collagen cross-linking, exacerbating ECM stiffness and fibrosis progression. LOXL2 inhibitors significantly alleviate D-galactose-induced aging-related muscle fibrosis and improve muscle mass and contractile function by suppressing the TGF-β1/p38 MAPK pathway (59). In summary, multi-target strategies that regulate ECM metabolism and remodeling—by promoting collagen degradation and inhibiting abnormal cross-linking—offer promising approaches for the clinical prevention and treatment of skeletal muscle fibrosis.

### 3.4 Targeting fibrosis-related cell populations

The development of skeletal muscle fibrosis is closely associated with the abnormal activation of multiple cell populations, particularly the activation and differentiation of FAPs and fibroblasts. Targeting these cell types has become a major strategy in anti-fibrotic therapy. One approach involves inhibiting the differentiation of FAPs into myofibroblasts to mitigate fibrosis progression. Studies indicate that the mechanosensitive transcriptional coactivators YAP/TAZ play a central role in TGF-β-induced fibrogenic transformation of FAPs. Inhibitors of YAP/TAZ, such as Verteporfin, or agents targeting the Rho pathway can effectively block mechanical signaling, thereby suppressing myofibroblast differentiation and collagen deposition (5). Additionally, the purinergic receptor P2Y2 promotes fibroblast proliferation and migration, and its antagonist AR-C118925 significantly inhibits fibroblast activation and excessive extracellular matrix production, reducing fibrotic responses (30). Another strategy involves regulating the degradation of key proteins to inhibit collagen production. For instance, in cancer cachexia-associated muscle fibrosis, l-carnitine upregulates the Deltex E3 ubiquitin ligase 3 L (DTX3L), promoting ubiquitination and degradation of the transcription factor Runx2. This subsequently inhibits transcription and protein synthesis of its downstream target gene COL1A1, ultimately reducing collagen deposition and ameliorating muscle fibrosis (4). In summary, interventions targeting the differentiation of FAPs and fibroblasts or modulating the stability of key fibrotic proteins offer multi-layered therapeutic strategies with broad translational potential for treating muscle fibrosis.

### 3.5 Gene and RNA-targeted therapeutic approaches

The molecular mechanisms underlying skeletal muscle fibrosis are complex and involve dysregulation of various RNA molecules. Targeting specific genes or RNAs thus demonstrates promising therapeutic potential. On one hand, restoring the expression of microRNAs (miRNAs) with anti-fibrotic functions—such as miR-29b, miR-133a, and miR-1—which are significantly downregulated in chronic limb-threatening ischemia (CLTI), can reduce the accumulation of extracellular matrix components like collagen. Furthermore, mesenchymal stromal cell (MSC) transplantation has been shown to ameliorate muscle ischemia and fibrosis, partly by restoring the expression of anti-fibrotic miRNAs like miR-29b, miR-1, and miR-133a, which target collagen genes (90). On the other hand, inhibiting pro-fibrotic miRNAs such as miR-21 and miR-199a-5p is essential. miR-21 promotes fibrosis by activating the TGF-β/SMAD pathway, while miR-199a-5p is delivered via exosomes from fibroblasts and exacerbates fibrotic responses (10, 37, 70). Additionally, long non-coding RNAs (lncRNAs), such as lnc-MFAT1, are highly expressed in fibrotic muscle. Its knockdown alleviates TGF-β-mediated fibrosis by competitively binding to miR-135a-5p, thereby reversing the suppression of TGFBR2/Smad4 (72). At the genetic level, local delivery of miRNA-based targeting systems—such as miR-AT1a directed against angiotensin II receptor type 1A (AT1a)—can significantly reduce receptor expression and downregulate TGF-β, Smad3, and collagen genes. This approach inhibits fibrosis specifically without affecting other organs (11). These strategies not only offer high specificity but also markedly improve the muscle regeneration microenvironment, presenting new directions for clinical treatment.

### 3.6 Biologics and cell therapy

The monoclonal antibody Pamrevlumab (FG-3019), which targets CTGF/CCN2, demonstrates significant anti-fibrotic effects in animal models. It reduces the expression of fibrosis-related factors such as TGF-β1, collagen I, and collagen III in muscle tissue and also restores muscle function, evidenced by improved grip strength. These findings suggest its potential in treating chronic overuse-induced muscle injury (91). On the other hand, MSCs, platelet-rich plasma (PRP), and their secretomes also show anti-fibrotic potential by modulating the inflammatory microenvironment, promoting angiogenesis, and supporting muscle fiber regeneration. However, their efficacy remains controversial. For example, local injection of PRP in a distraction osteogenesis model did not significantly reduce collagen deposition or alleviate muscle fibrosis (92). Although such biologic agents act through multiple mechanisms to regulate extracellular matrix homeostasis, their clinical application requires further high-quality studies for validation (93). Overall, these therapeutic strategies provide diverse research directions for intervening in muscle fibrosis, but more evidence is needed to facilitate their translation into clinical practice.

### 3.7 Physical therapy and nutritional interventions

In the prevention and treatment of skeletal muscle fibrosis, physical therapies and nutritional interventions demonstrate multi-faceted therapeutic potential (94). Combined electroacupuncture and massage therapy can mitigate fibrosis by modulating the expression of key factors. Studies have shown that this combined approach significantly suppresses the mRNA and protein expression of TGF-β1 and CTGF. This reduction further decreases myofibroblast transdifferentiation and excessive extracellular matrix deposition. Additionally, it helps rebalance MMP-1 and TIMP-1, promoting collagen degradation and improving muscle architecture (89). Furthermore, percutaneous needle fasciotomy (PNF) combined with stretching has emerged as a superior physical intervention compared to acupuncture alone in alleviating early-stage skeletal muscle fibrosis in rats, effectively modulating the TGF-β1/Smad pathway (95). Furthermore, magnetic stimulation therapy, which induces muscle twitch contractions, has been shown to alleviate fibrosis and restricted joint mobility caused by immobilization. This treatment significantly downregulates the expression of fibrosis-related genes such as type I and III collagen, HIF-1α, and α-SMA. It also reduces hydroxyproline content in muscle, thereby improving fibrosis and functional recovery (96). Regarding nutritional interventions, natural active compounds like geniposide—extracted from gardenia fruit residue—exhibit notable anti-fibrotic effects. Its mechanism primarily involves inhibiting the TGF-β/Smad4 signaling pathway, leading to significant downregulation of multiple pro-fibrotic genes including collagen. It also ameliorates muscle tissue damage in contusion models (97, 98). Sodium tanshinone IIA sulfonate, a traditional Chinese medicine compound, also ameliorates skeletal muscle injury and fibrosis by dually inhibiting the TGF-β1/Smad3 and PI3K/Akt/COX-2 signaling pathways (99). In summary, physical modalities such as electroacupuncture and magnetic stimulation, along with supplementation of specific natural products, offer multi-target and comprehensive strategies for the clinical prevention and treatment of skeletal muscle fibrosis.

### 3.8 Combination therapies and future directions

The pathogenesis of skeletal muscle fibrosis is complex and involves interactions among multiple cytokines, signaling pathways, and cell types. Research indicates that TGF-β plays a central role in promoting myofibroblast transdifferentiation and extracellular matrix deposition (89). Mechanical stimuli such as muscle twitch contractions can alleviate fibrosis to some extent and improve joint mobility by regulating hypoxia-inducible factor 1α (HIF-1α), α-smooth muscle actin (α-SMA), and collagen expression (96). Additionally, natural compounds like geniposide, a component of traditional Chinese medicine, significantly reduce the expression of pro-fibrotic genes by inhibiting the TGF-β–Smad4 signaling pathway, suggesting their potential in anti-fibrotic therapy (97). Thus, multi-target intervention strategies may offer new directions for treating skeletal muscle fibrosis.

Given the multifactorial nature of fibrotic pathogenesis, single therapies often show limited efficacy. Multi-target combination strategies have gradually become a research focus. For example, ACEIs combined with exercise training synergistically improve muscle blood flow and the fibrotic microenvironment (32). Anti-TGF-β agents combined with antioxidants can simultaneously inhibit pro-fibrotic signaling and oxidative stress damage (100). Meanwhile, YAP/TAZ inhibitors in the Hippo pathway combined with the regulation of FAPs may intervene in fibrosis through both mechanotransduction and cell fate determination (44). Therefore, multi-target synergistic strategies, by simultaneously intervening in different facets of fibrosis, demonstrate significant therapeutic potential, though their clinical translation requires further validation and optimization.

For efficacy evaluation, traditional histological methods are invasive and unsuitable for dynamic monitoring. Emerging imaging techniques such as shear wave elastography (SWE) enable non-invasive and quantitative assessment of muscle stiffness and fibrosis severity, providing a powerful tool for objective quantification of treatment outcomes (101). In summary, treating fibrosis requires multi-target and multimodal strategies combined with advanced imaging for dynamic assessment to achieve personalized precision medicine.

## 4 Discussion and outlook

Current therapeutic strategies for skeletal muscle fibrosis have become increasingly diverse, encompassing molecular targeting, cellular regulation, physical therapy, and nutritional supplementation. Studies indicate that targeting key signaling pathways such as TGF-β/Smad and YAP/TAZ or modulating functional cells including FAPs and macrophages can significantly reduce fibrosis and improve muscle function (1, 5). Furthermore, several natural compounds such as highland barley tea polyphenols and irisin as well as existing drugs including SGLT2 inhibitors and ACE inhibitors have demonstrated promising anti-fibrotic potential (5, 62, 102). Physical interventions like electroacupuncture combined with massage or lumbar magnetic stimulation to induce contractions can alleviate fibrosis by modulating local inflammation and extracellular matrix metabolism (89, 96, 103). These advances provide a critical foundation for developing comprehensive treatment approaches.

Despite abundant preclinical research, most interventional strategies remain at the animal experimentation stage, and their translation into clinical practice faces multiple challenges. First, there is significant disease heterogeneity. Fibrosis triggered by different etiologies—such as DMD, aging-related sarcopenia, cancer cachexia, and diabetes—varies in molecular mechanisms and pathological features (1, 4, 104). For instance, DMD is characterized by excessive activation of FAPs and enhanced collagen cross-linking, while cancer cachexia is closely associated with RUNX2/COL1A1 axis activation (5, 15). Therefore, developing personalized treatment strategies is particularly necessary.

Second, the timing and dosage of treatment significantly influence therapeutic efficacy. Studies indicate that the early stages of fibrosis respond better to pharmacological intervention. For example, early administration of TGF-β trap proteins or ACE inhibitors after muscle injury can significantly delay fibrosis progression (78, 81). In contrast, late-stage fibrotic tissue exhibits structural remodeling and a solidified microenvironment, which greatly increases treatment difficulty.

Third, given that fibrosis involves complex interactions among multiple signaling pathways and cell types, single-target therapies often yield limited effects. Combination strategies—such as pairing TGF-β inhibitors with antioxidants like astaxanthin, or combining anti-inflammatory and pro-regenerative approaches—show synergistic potential (5, 59). For example, simultaneous inhibition of TGF-β and CCN2 has been shown to markedly reverse fibrosis and restore muscle function across multiple models (91). Thus, multi-target combination therapies may represent a key direction for future anti-fibrotic drug development.

Finally, several bottlenecks hinder clinical translation. Existing animal models do not fully recapitulate the complexity and chronic progression of human diseases, and caution is warranted when using mdx mice to study DMD (11, 12). Moreover, the safety profiles and long-term side effects of treatments require further validation through extensive clinical data.

Looking ahead, research should focus on developing highly specific and low-toxicity targeted therapies, such as small molecule inhibitors or antibodies against emerging targets like YAP/TAZ, LOXL2, and Galectin-3 (5, 9, 59). In parallel, the integration of imaging techniques (e.g., ultrasound elastography and MRI) and liquid biopsies may help identify early biomarkers, enabling early diagnosis and dynamic monitoring of treatment response (10, 101). The application of multi-omics and spatial transcriptomics will further elucidate cellular heterogeneity and molecular networks underlying fibrosis from diverse causes, providing a foundation for precision medicine. Ultimately, through deeper integration of basic and clinical research, personalized treatment strategies may emerge as a promising approach against skeletal muscle fibrosis.
